# Development and validation of artificial intelligence models for early detection of postoperative infections (PERISCOPE): a multicentre study using electronic health record data

**DOI:** 10.1016/j.lanepe.2024.101163

**Published:** 2024-12-05

**Authors:** Siri L. van der Meijden, Anna M. van Boekel, Laurens J. Schinkelshoek, Harry van Goor, Ewout W. Steyerberg, Rob G.H.H. Nelissen, Dieter Mesotten, Bart F. Geerts, Mark G.J. de Boer, M. Sesmu Arbous, PERISCOPE Group, Pieter de Heer, Jaap Hamming, Karin Ellen Veldkamp, Wilco Peul, Rolv-Ole Lindsetmo, Maxime Kummeling, Jogchum Beltman, Merlijn Hutteman, Alma Tostman, Wim Reijnen, Bas Bredie, Ilse Spenkelink, Ben Goethuys, Noëlla Pierlet, Joost Huiskens

**Affiliations:** a Intensive Care Unit, Leiden University Medical Centre, Leiden, the Netherlands; b Healthplus.ai B.V., Amsterdam, the Netherlands; c General Surgery Department, Radboud University Medical Centre, Nijmegen, the Netherlands; d Department of Biomedical Data Sciences, Leiden University Medical Centre, Leiden, the Netherlands; e Department of Orthopaedics, Leiden University Medical Centre, Leiden, the Netherlands; f Department of Anaesthesiology, Intensive Care Medicine, Ziekenhuis Oost-Limburg, Genk, Belgium; g Faculty of Medicine and Life Sciences, Limburg Clinical Research Centre, UHasselt, Diepenbeek, Belgium; h Department of Infectious Diseases, Leiden University Medical Centre, Leiden, the Netherlands

**Keywords:** Postoperative infection, Artificial intelligence, Multi-centre validation, Model updating, Clinical utility

## Abstract

**Background:**

Postoperative infections significantly impact patient outcomes and costs, exacerbated by late diagnoses, yet early reliable predictors are scarce. Existing artificial intelligence (AI) models for postoperative infection prediction often lack external validation or perform poorly in local settings when validated. We aimed to develop locally valid models as part of the PERISCOPE AI system to enable early detection, safer discharge, and more timely treatment of patients.

**Methods:**

We developed and validated XGBoost models to predict postoperative infections within 7 and 30 days of surgery. Using retrospective pre-operative and intra-operative electronic health record data from 2014 to 2023 across various surgical specialities, the models were developed at Hospital A and validated and updated at Hospitals B and C in the Netherlands and Belgium. Model performance was evaluated before and after updating using the two most recent years of data as temporal validation datasets. Main outcome measures were model discrimination (area under the receiver operating characteristic curve (AUROC)), calibration (slope, intercept, and plots), and clinical utility (decision curve analysis with net benefit).

**Findings:**

The study included 253,010 surgical procedures with 23,903 infections within 30-days. Discriminative performance, calibration properties, and clinical utility significantly improved after updating. Final AUROCs after updating for Hospitals A, B, and C were 0.82 (95% confidence interval (CI) 0.81–0.83), 0.82 (95% CI 0.81–0.83), and 0.91 (95% CI 0.90–0.91) respectively for 30-day predictions on the temporal validation datasets (2022–2023). Calibration plots demonstrated adequate correspondence between observed outcomes and predicted risk. All local models were deemed clinically useful as the net benefit was higher than default strategies (treat all and treat none) over a wide range of clinically relevant decision thresholds.

**Interpretation:**

PERISCOPE can accurately predict overall postoperative infections within 7- and 30-days post-surgery. The robust performance implies potential for improving clinical care in diverse clinical target populations. This study supports the need for approaches to local updating of AI models to account for domain shifts in patient populations and data distributions across different clinical settings.

**Funding:**

This study was funded by a REACT EU grant from 10.13039/501100008530European Regional Development Fund (ERDF) and Kansen voor West.

**Research in context**

**Evidence before this study**

Predictive models and biomarkers have been previously developed and studied to assist in the early diagnosis and detection of different types of postoperative infections. We searched Medline, Embase, and the Cochrane database for articles published in English between inception and January 1, 2024, using search terms “prediction”, “decision support”, “artificial intelligence”, “biomarker”, “model”, “postoperative infection”, and synonyms. We identified 104 relevant studies describing the performance of 14 different AI models, 7 biomarkers, and 45 statistical models. Most studies reported only internal validation results on specific patient populations and specific types of infections. There was a relatively lack of multi-centre, external validations of both classic prediction models (e.g., the American College of Surgeons National Surgical Quality Improvement Program (ACS NSQIP)) and AI models. None of the studies performed local model updates in a multicentre setting to investigate the impact on discriminative performance, calibration, and clinical utility.

**Added value of this study**

To our knowledge, this is the first study reporting on the development and external validation of AI models to predict the risk of overall postoperative infections across diverse patient populations in a multi-centre setting in two countries. By employing an extensive validation and updating approach using local patient data, we contributed to a roadmap for the implementation of locally valid AI systems in the future. We showed that discriminative performance, calibration, and clinical utility (net benefit) improved after updating per site. The improvement after local updating may be attributed to domain shift, i.e., differences in case mix, incidence, treatment pathways, and data collection methods. Local models achieved AUROCs ≥ 0.82. A thorough subgroup analysis was conducted to assess biases across different demographics and patient groups. The results of this study align with recent commercial and governmental attention to local validations and monitoring of performance in subgroups.

**Implications of all the available evidence**

To enhance the implementation of locally valid AI systems in clinical practice, we present a framework allowing the safe scaling of locally updated AI models. Supported by the data from this study, we advocate not to develop and use “one-size-fits-all” products but include local validation and updating in AI systems because of differences between patient populations across hospitals. By following our methodology, we developed the AI system PERISCOPE to predict overall postoperative infections with high performance in terms of discrimination, calibration, and clinical utility. Clinical trials are planned to investigate the impact on patient outcomes and costs.

## Introduction

Postoperative bacterial infections are an important concern in modern healthcare, with major implications for patient well-being and healthcare costs.^1^ These infections, ranging from surgical site infections (SSIs) to systemic complications like sepsis, occur in 6.5–18.3% of surgical patients, classifying them amongst the most common postoperative complications.^2^, ^3^, ^4^, ^5^ These infections affect the 313 million global surgeries performed annually as well as the 150 million patients who do not receive their necessary surgeries because of competition for hospital capacity and operating room time.^6^ Postoperative infections extend hospital stays by 20–50%, increase morbidity and mortality rates,^7^^,^^8^ and account for up for most unplanned hospital readmissions within 30-days of surgery.^9^ The significant impact of these infections has driven efforts to improve diagnostic and predictive systems to mitigate these risks and enhance patient outcomes.

Artificial intelligence (AI) brings the promise of early identification and detection of postoperative infections.^10^, ^11^, ^12^ However, AI systems, including the model, user interface, and information technology (IT) integration,^13^ are rarely used outside radiology and pathology diagnostics due to fundamental problems like data standardisation, certification-related issues, and lack of technical expertise.^14^ For previous applications that did reach the implementation stage, real-life predictive performance often severely decreased.^15^ This decline in performance highlights the need for extended validations and updating of models to achieve acceptable performance in specific target populations.^16^ The drop in external model performance in AI models may be attributed to a large number of input parameters and model flexibility, making them sensitive to variations in input data and patient populations.^17^^,^^18^ Over 80% of studies on AI in surgery report only internal validation results with small sample sizes, lacking performance data across different sociodemographic groups, leading to bias and patient fairness issues.^19^ The absence of external validations in specific clinical target populations hampers safe implementation of predictive models due to differences in case mix, incidence, treatment pathways, and data collection methods, resulting in poor performance.^16^ This change in data distributions between development and external hospital datasets is referred to as a “domain shift”.^20^ Therefore, ‘off-the-shelf’ models without validation in a local setting before implementation and, if needed, local updates may not be suitable for clinical decision support.

We aimed to develop locally valid postoperative infection predictive models to assist early detection of a postoperative infection, one of the most frequent surgical complications after surgery. We hereto present the multi-centre development and validation of the predictive models in the AI system PERISCOPE® that is integrated into the electronic health record (EHR) system to predict the probability of developing a postoperative infection for a wide range of surgical procedures. PERISCOPE provides a short-term (within 7 days of surgery) and a longer-term (within 30 days of surgery) prediction. The prediction is issued within 1 h after the procedure is completed and is intended to be used by physicians and nurses in the postoperative setting. First, we developed XGBoost models at the development site. XGBoost is an optimised gradient boosting machine learning algorithm known for its efficiency and predictive power.^21^ Secondly, we externally validated these models at two international sites, evaluating their performance in terms of discrimination, calibration, and clinical utility. Thirdly, we used local patient data from the validation sites to create updated models. Fourthly, we assessed whether these updated versions of PERISCOPE achieved higher performance. Lastly, the final models underwent extensive subgroup analyses at each hospital site.

## Methods

### Study design and participants

Patients from two Dutch and one Belgian hospital were included in model development and validation. Pre-operative and intra-operative routine care EHR data were collected from surgical patients. The development site was the Leiden University Medical Centre (Hospital A), and the external validation and updating sites were the Radboud University Medical Centre (Hospital B), and Hospital Oost-Limburg Genk (Hospital C). Patients were included in the dataset more than once if they underwent multiple procedures separated by more than 30 days. The unit of analysis was, therefore, the individual surgical procedures performed. Excluded from training and prediction were procedures of paediatric patients (age <18 years), patient admissions with the primary procedures being to treat an infection, cardiological or radiological interventions, electroshock-, radiation-, or brachytherapy, diagnostic endoscopy, taking of a biopsy, eye surgery and procedures on pregnant patients. See the Supplementary Materials Section 2 for sample size calculations. All datasets achieved the required sample sizes, being more than 10,000 procedures for the development and updating datasets and more than 2000 procedures for the validation datasets.

This study was performed in compliance with the Declaration of Helsinki. The study protocol was reviewed and waived for medical ethical committee approval by the Leiden University Medical Centre, Leiden, the Netherlands (**G18**.**129**) according to the Dutch and Belgium law. All data were coded (pseudo-anonymised). The Transparent Reporting of a Multivariable Prediction Model for Individual Prognosis Or Diagnosis (TRIPOD-AI) guidelines were adhered to when writing this manuscript.^22^ There was no patient or public involvement during this study.

### Ground-truth labelling

A broad definition was used to capture all types of bacterial postoperative infections, including SSIs, urinary tract infections, respiratory tract infections, and others. Infectious complications are known to be under-reported based on different definitions in clinical patient records, and manual chart review of all patient records was not feasible due to the size of the datasets.^23^^,^^24^ Therefore, we considered a clinically relevant label definition including all registered infections and infections for which patients received pharmacological or surgical treatment. Based on this definition, infectious complications were labelled with a Clavien-Dindo score of 1–3.^25^ Labelling was done as part of the data pre-processing using complication registries, medication administrations, and procedure data. A procedure was labelled positive for the 7-day and 30-day outcome if 1) an infectious complication was registered, 2) antibiotics were administered for more than 72 h, excluding prophylactic regimes, and/or 3) any surgical intervention was performed to treat an infection for the relevant time frame. See Supplementary Materials Section 2.3 for more details on the labelling process.

### Data partition

The dataset from Hospital A was divided into a development dataset (years 2014–2021) and a temporal validation dataset (years 2022–2023). Datasets from Hospital B and C were each divided into an updating dataset (respectively years 2014–2021 and 2018–2021) and a temporal validation dataset (years 2022–2023). The performance of the models developed on Hospital A (years 2014–2021) was first analysed on the validation datasets from Hospitals B (years 2022–2023) and C (years 2022–2023) to ensure a fair comparison between pre- and post-updated models (Table A1, Supplementary Materials). Years 2022–2023 were used per site as a validation dataset to evaluate stable performance over time. Ten-fold cross-validation was performed at each site, using 80% of the respective development or updating dataset for model training and 20% for testing.

### Variables and features

A selection of 56 input variables was used, including vital functions, patient characteristics, laboratory results, procedure characteristics, preoperative anaesthesia questionnaires (e.g., ASA score), comorbidities, and medication history. See the Supplementary Materials Section 2.2, Table A3. These features were chosen based on literature review on risk factors for postoperative infection and included if available in the EHR databases. Time-series data were aggregated for each procedure across two time periods: 1) 24 h before surgery and 2) during surgery, with metrics such as the mean and maximum heart rate calculated for both periods. Categorical features were one-hot encoded. It was investigated whether features were missing at random or not at random by assessing the difference in missingness between the patient groups with the outcome and without the outcome. If no significant difference was found, missingness was determined to be random and median imputation was performed. See the Supplementary Materials Section 2.4 for the full imputation strategy. Data balancing strategies were not applied as this often leads to miscalibration.^26^

### Model development

XGBoost machine learning models were trained on the Hospital's A development dataset to predict the probability between 0 and 100% of developing a postoperative infection within 7 days and 30 days of surgery.^27^ During training of XGBoost models, ensembles of decision trees are built and iteratively improved by minimising the prediction errors of previous trees using gradient descent.^21^ XGBoost was chosen as it outperformed other machine learning models in terms of discriminative performance.^28^^,^^29^ Hyperparameters of the 30-day and 7-day prediction models were tuned separately in Python version 3.8 Models were optimised for the Area Under the Receiver Operating Characteristic curve (AUROC). For additional pre-processing and hyperparameter details, see the Supplementary Materials Sections 2.2 and 2.5. To determine if the choice of development site affected the need for model updating, we experimented with using Hospitals B and C as alternative development sites. Models developed from each were then validated using the other two hospitals as external validation sites. See Supplementary Materials, Figure A2 for an example of the PERISCOPE dashboard. PERISCOPE was designed to provide healthcare professionals with additional information to support decisions regarding the intensification of monitoring or supporting discharge decisions. It does not currently recommend or predict specific actions based on its predictions and leaves the final decisions to the healthcare professionals, who use their clinical judgement alongside the model's predictions. Local protocols may be adapted in response to experience with using PERISCOPE, but these decisions ultimately fall under the purview of the speciality or department's clinical leadership. Specifically, a high risk of infection in a presumed low-risk surgery may imply prolonged monitoring. Conversely, negligible risk of infection in a presumed high-risk surgery may suggest an earlier discharge.

### Model updating

The predictive models implemented in the PERISCOPE AI system were developed on Hospital A's development dataset, and externally validated on the temporal validation datasets (years 2022–2023, Table A1, Supplementary Materials). The model development at site A involved feature selection and determining the hyperparameter tuning approach, and building the machine learning pipeline including data preprocessing, hyperparameter tuning, and model training. This same machine learning pipeline including the same set of features was applied at sites B and C. Updating included hyperparameter tuning and retraining on local updating datasets, and optionally recalibration if the calibration was not adequate after retraining alone.^22^ After external validation and updating, the highest-performing models were used for further evaluation, referred to as the ‘final’ models. This resulted in two final models per site, one XGBoost model for the prediction of postoperative infections within 7 days of surgery and one XGBoost model for the prediction within 30 days of surgery. An extensive subgroup analysis was performed to assess for biases based on sex as reported by physicians, age, surgery priority, surgical speciality, surgical procedure location, type of surgery (open or laparoscopic), admission type, and whether the patient had an ongoing infection at the moment of surgery. No race, ethnicity, and socioeconomic status data were available for additional fairness analyses. We evaluated biases in subgroups in terms of discriminative performance, calibration properties, and clinical utility by means of decision curve analysis (net benefit) as explained in the following section. SHapley Additive exPlanations (SHAP) values were assessed to investigate model explainability and differences in predictive features that could explain domain shift across sites.^30^ The code to generate the performance metrics and subgroup analyses is available at Gitlab.^31^ The machine learning pipeline for PERISCOPE is proprietary and therefore not available for public reuse.

### Statistical analysis

Descriptive characteristics were presented per site with absolute number and percentage, mean and standard deviation or median with interquartile range where appropriate. Model performance was evaluated in terms of discriminative performance, calibration, and clinical utility.^32^ *Discriminative* performance metrics assessed included sensitivity, specificity, positive predictive value (PPV), negative predictive value (NPV), accuracy, and F1 score (harmonic mean of PPV and sensitivity), and are reported using the incidence of postoperative infections in the development dataset or updating dataset per site as a classification threshold. Threshold independent metrics evaluated included AUROC and area under the precision-recall curve (AUCPR). AUROC is calculated by plotting the sensitivity against the false positive rate (1-specificity) at various thresholds and measuring the area under this curve. AUCPR is calculated similarly by plotting precision (PPV) against recall (sensitivity). *Calibration* plots were assessed with calibration slope and intercept. These plots show the agreement between the predicted probabilities and the proportion of outcomes in patients with these predictions. Perfectly calibrated models have a slope of 1 and an intercept of 0. Adequate calibration is determined as no systematic deviations are visible in the calibration plots.^32^ To evaluate PERISCOPE beyond its discriminative and calibration properties, the *clinical utility* was calculated using decision curve analysis. Net benefit was assessed for a range of clinically relevant decision thresholds, as it may differ per patient and type of intervention how many false positives are accepted to find one true positive.^33^ The unit of net benefit is ‘true positives’ At lower decision thresholds, the end-user is more worried about the disease, i.e., willing to accept more false positives, and at higher decision thresholds, the end-user is more worried about unnecessary interventions following the prediction.^34^ To be of clinical value, net benefit should be higher than zero and default strategies in the established range of clinically relevant risk thresholds. We considered net benefit for thresholds between 0 and 30%. The six final models (7-day and 30-day models for Hospitals A, B, and C) were evaluated on all hospitals' temporal validation datasets using bootstrapping (1000 samples with replacement) to assess 95% confidence intervals. Subgroups needed to meet the minimum required sample size to be evaluated. All statistical analyses were performed in Python version 3.8 and R version 4.1.2. A study protocol was created as part of the clinical evaluation of PERISCOPE and is available upon request.

### Role of the funding source

This study was funded by a “REACT EU” grant from “European Regional Development Fund (ERDF)” and “Kansen voor West” with grant number KVW-00351. The funders had no role in study design, data collection, data analysis, interpretation, or manuscript writing.

## Results

Out of 574,831 identified surgical procedures, 253,010 with a total of 9.4% (n = 23,903) 30-day infections from 210,247 unique patients were included. We note that 158,989 procedures were excluded for being non-invasive (e.g., anaesthesiologic and endoscopic procedures) and 162,832 procedures for meeting the other exclusion criteria (Supplementary Materials Figure A1). Summary statistics and procedure characteristics were compared between development (Hospital A, years 2014–2021) or updating (Hospital B, years 2014–2021, and C, years 2018–2021) and temporal validation datasets (all hospitals, years 2022–2023, Table 1). Electronic health record data were processed for all included procedures and labelled for the predicted outcome of interest according to the predefined outcome. The overall postoperative infection rates within 30 and 7 days of surgery for Hospital A were respectively 1489/10,705 14% and 829/10,705 (8%), for Hospital B 1987/13,474 (14%) and 987/13,474 (7%), and for Hospital C 1889/50,230 (4%) and 1260/50,230 (3%). Hospital C was the only non-academic hospital and had a higher proportion of orthopaedic surgeries and lower ASA scores, which are correlated with lower infection rates.

**Table 1:** Patient characteristics for development (Hospital A), updating (Hospitals B and C), and validation datasets.

	Hospital A		Hospital B		Hospital C	
	Development dataset (2014–2021)	Validation dataset (2022–2023)	Updating dataset (2014–2021)	Validation dataset (2022–2023)	Updating dataset (2018–2021)	Validation dataset (2022–2023)
**Number of procedures (%)**	46,770 (81.4)	10,705 (18.6)	58,575 (81.3)	13,474 (18.7)	69,410 (58.0)	50,230 (42.0)
**7 day infections (%)**	3992 (8.5)	829 (7.7)	4272 (7.3)	987 (7.3)	2095 (3.0)	1260 (2.5)
**30 day infections (%)**	6915 (14.8)	1489 (13.9)	8466 (14.5)	1987 (14.7)	3057 (4.4)	1889 (3.8)
**Median age (IQR)**	58 (45–69)	59 (44–70)	59 (45–69)	61 (45–71)	56 (41–68)	57 (42–69)
**Male sex (%)**	22,307 (47.7)	4997 (46.7)	30,305 (51.7)	6971 (51.7)	32,324 (46.6)	23,786 (47.4)
**Mean BMI (std)**	26.1 (4.98)	26.2 (5.02)	26.3 (4.84)	26.5 (4.91)	26.8 (5.16)	27.0 (5.18)
**Procedure duration in minutes (IQR)**	90 (46–156)	95 (48–164)	88 (43–157)	96 (48–173)	80 (50–123)	74 (46–113)
**Surgical specialities**						
Gynaecology	4054 (8.7)	990 (9.2)	6034 (10.3)	1455 (10.8)	5053 (7.3)	3304 (6.6)
Plastic surgery	1822 (3.9)	272 (2.5)	N/A	N/A	2123 (3.1)	1783 (3.5)
Vascular surgery	N/A	N/A	N/A	N/A	4425 (6.4)	3362 (6.7)
Maxillofacial surgery	1568 (3.4)	354 (3.3)	4222 (7.2)	1034 (7.7)	4007 (5.8)	2390 (4.8)
Otolaryngology surgery	6635 (14.2)	1578 (14.7)	6398 (10.9)	1260 (9.4)	4227 (6.1)	2598 (5.2)
Neurosurgery	5943 (12.7)	1694 (15.8)	6482 (11.1)	1643 (12.2)	7410 (10.7)	4386 (8.7)
Orthopaedic surgery	5113 (10.9)	965 (9.0)	9162 (15.6)	1945 (14.4)	23,576 (34.0)	18,409 (36.6)
General surgery	18,410 (39.4)	4225 (39.5)	9179 (15.7)	2040 (15.1)	13,440 (19.4)	10,019 (19.9)
Urology	3225 (6.9)	627 (5.9)	9218 (15.7)	2082 (15.5)	3586 (5.2)	3279 (6.5)
Thorax surgery	N/A	N/A	7880 (13.5)	2015 (15.0)	1563 (2.3)	700 (1.4)
**Procedure urgency**						
Elective	37,508 (80.2)	8547 (79.8)	50,716 (86.6)	11,571 (85.9)	62,683 (90.3)	45,229 (90.0)
As soon as possible	2435 (5.2)	1848 (17.3)	6068 (10.4)	1549 (11.5)	5128 (7.4)	4183 (8.3)
Emergency	6827 (14.6)	310 (2.9)	1791 (3.1)	354 (2.6)	1599 (2.3)	818 (1.6)
**Median ASA score (IQR)**	2 (2–2)	2 (2–3)	N/A	N/A	2 (1–2)	2 (1–2)

Values are reported with absolute number and percentage, mean and standard deviation or median with interquartile range where appropriate.

ASA = American Society of Anaesthesiologists. BMI = Body mass index. IQR = Interquartile range.

The models developed on Hospital A's development dataset were first validated on Hospital A's validation dataset (AUROC 30-day 0.82 (0.81–0.83), 7-day 0.81 (0.79–0.82)). Models were afterwards externally validated on the temporal validation datasets from Hospital B (AUROC 30-day 0.77 (0.76–0.79), 7-day 0.77 (0.75–0.79)) and Hospital C (AUROC 30-day 0.85 (0.84–0.86), 7-day 0.86 (0.85–0.87)). After the models were updated on historical and local data (Table 2), discriminative performance improved for both Hospital B (AUROC 30-day 0.82 (0.81–0.83), 7-day 0.81 (0.80–0.83)) and Hospital C (AUROC 30-day 0.91 (0.90–0.91), 7-day 0.92 (0.91–0.92)). Alternating the development site to Hospital B or Hospital C resulted in similar improvements after updating (Supplementary Materials Section 1, Table A2). Additional performance metrics for the final, locally updated models are presented in the Supplementary Materials (Tables A8–A10). The AUCPR for the final models ranged between 0.26 and 0.51.

**Table 2:** Discriminative performance (AUROC) and calibration slope and intercept of PERISCOPE 30-day and 7-day predictive models before and after updating.

Prediction timeframe	Metric	Hospital A	Hospital B		Hospital C	
		Validation dataset performance	Validation dataset (before updating)	Validation dataset (after updating)	Validation dataset (before updating)	Validation dataset (after updating)
30 days	AUROC (95% CI)	**0.82 (0.81–0.83)**	0.77 (0.76–0.79)	**0.82 (0.81–0.83)**	0.85 (0.84–0.86)	**0.91 (0.90–0.91)**
30 days	Calibration slope (95% CI)	**0.90 (0.85–0.94)**	0.89 (0.85–0.94)	**0.94 (0.90–0.98)**	1.90 (1.85–1.96)	**0.95 (0.92–0.98)**
30 days	Calibration intercept (95% CI)	**−0.10 (−0.16 to 0.03)**	−0.41 (−0.45 to −0.36)	**−0.06 (−0.12 to 0.00)**	−1.08 (−1.12 to −1.04)	**−0.02 (−0.07 to 0.04)**
7 days	AUROC (95% CI)	**0.81 (0.79–0.82)**	0.77 (0.75–0.79)	**0.81 (0.80–0.83)**	0.86 (0.85–0.87)	**0.92 (0.91–0.92)**
7 days	Calibration slope (95% CI)	**0.85 (0.80–0.91)**	0.95 (0.89–1.01)	**0.95 (0.90–1.01)**	1.90 (1.83–1.96)	**0.93 (0.90–0.97)**
7 days	Calibration intercept (95% CI)	**−0.13 (−0.20 to −0.05)**	−0.48 (−0.54 to −0.41)	**−0.03 (−0.10 to 0.04)**	−0.43 (−0.47 to −0.38)	**−0.01 (−0.07 to 0.06)**

Results are shown on each hospital's temporal validation dataset (years 2022–2023).

Bold = local model performance. 95% confidence intervals were calculated using bootstrapping (1000 samples with replacement).

AUROC = Area under the receiver operating characteristic curve. CI = Confidence interval.

**Fig. 1:**
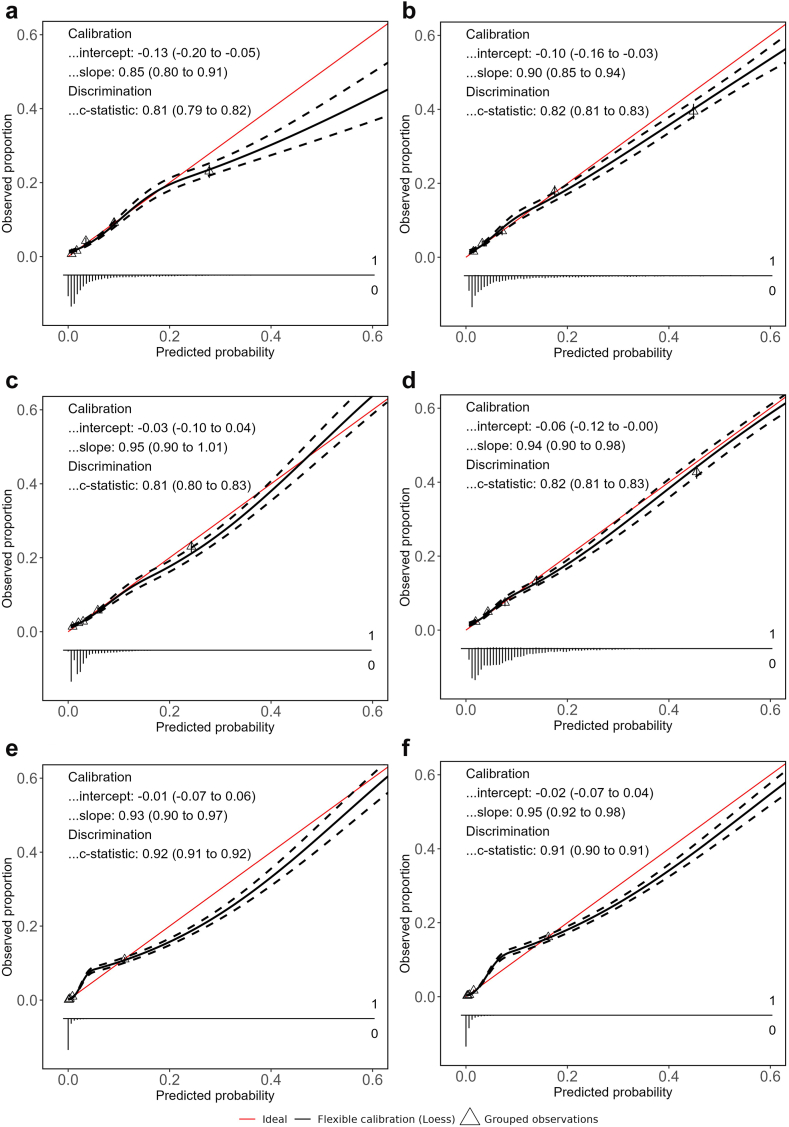
Calibration plots of final, locally updated models on the validation datasets (years 2022–2023): **a**) Hospital A – 7 day predictions, **b**) Hospital A – 30 day predictions, **c**) Hospital B – 7 day predictions, **d**) Hospital B – 30 day predictions, **e**) Hospital C – 7 day predictions, and **f**) Hospital C – 30 day predictions. The calibration curves (upper plots) show the agreement between the predicted probabilities by the model and the outcome of patients for those predictions. The histograms in the lower plot show the distributions of predictions.

**Fig. 2:**
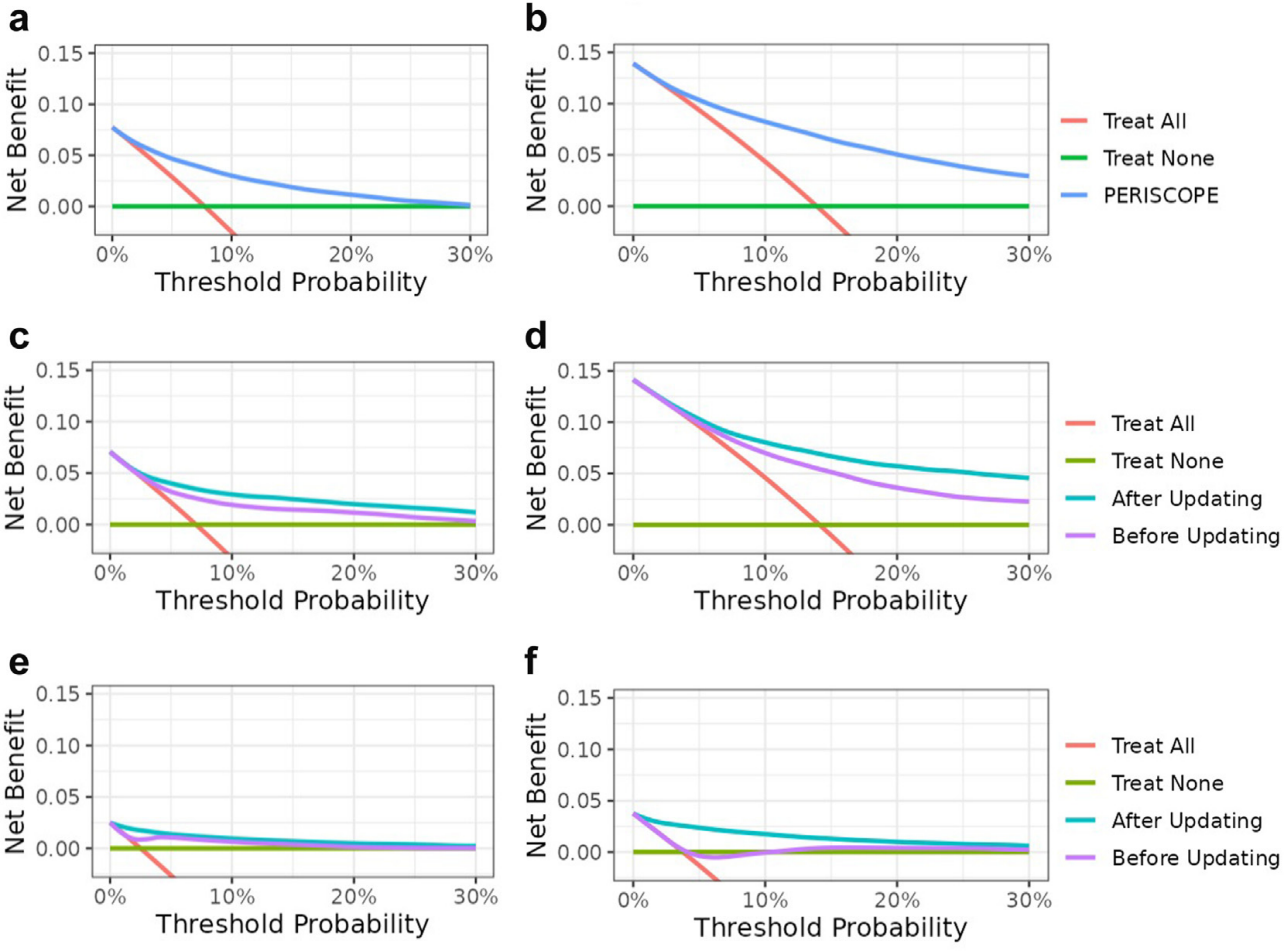
Decision curves showing the net benefit for PERISCOPE's models before and after updating compared to ‘treat all’ and ‘treat none’ patients for validation datasets (years 2022–2023): **a**) Hospital A – 7 day predictions, **b**) Hospital A – 30 day predictions, **c**) Hospital B – 7 day predictions, **d**) Hospital B – 30 day predictions, **e**) Hospital C – 7 day predictions, and **f**) Hospital C – 30 day predictions. The unit of net benefit is ‘true positives’. At lower decision thresholds, the end-user is more worried about the disease, i.e., willing to accept more false positives, and at higher decision thresholds, the end-user is more worried about the intervention following the prediction. To be of clinical value, net benefit should be higher than zero and default strategies in the established range of clinically relevant risk thresholds.

Calibration slopes and intercepts improved after updating (Table 2). Calibration curves (Fig. 1) for the final, locally updated models showed good calibration in the lower prediction region, with minimal overestimation (slopes ranging between 0.85 and 0.95) in all three sites. The calibration slope was smaller than 1, implying weaker overall associations of predictors with infections. Hospital C showed a slight underestimation in the region of the incidence rate. Overestimation increased in the higher prediction ranges (>30%) (Fig. 1, top right panel), but remained close to the ideal line in the lower ranges, where most predictions occurred. Therefore, no further recalibration was performed as part of the updating procedure. Decision curve analysis showed a positive net benefit compared to ‘treat all’ and ‘treat none’ strategies for all models, as well as an improved net benefit for locally updated models (Fig. 2), indicating increased clinical utility. Due to differences in postoperative infection rates, the net benefit curve crosses the y-axis at different points equal to the infection rates. Final model specifications with corresponding (hyper)parameters are part of the CE-certified PERISCOPE AI system and remain proprietary.

**Fig. 3:**
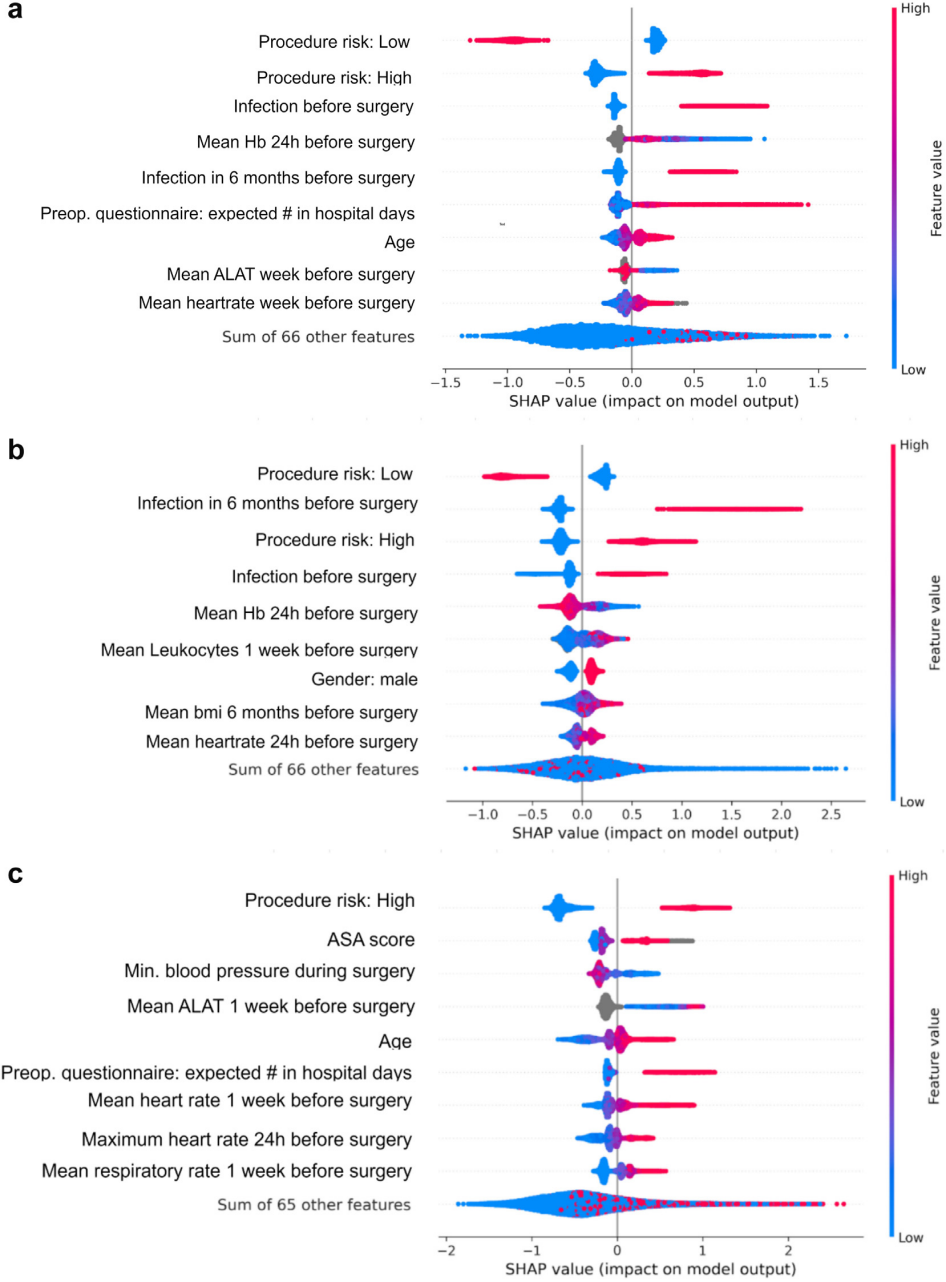
Model explainability in terms of SHAP (SHapley Additive exPlanations) values for: **a**) Hospital A – 30 day predictions, **b**) Hospital B – 30 day predictions, and **c**) Hospital C – 30 day predictions. For Hospitals B and C, updated models were used for predictions. In the SHAP plot, red dots represent high feature values and blue dots represent low feature values. Dots on the right side of the y-axis indicate a contribution to a higher predicted risk of infection, while dots on the left side indicate a lower risk. Grey dots signify missing feature values.

An extensive subgroup analysis was performed on groups meeting the minimum determined sample size in each validation dataset to assess biases and subgroup performance for the updated 30-day models (Supplementary Materials Tables A11–A13). Subgroup analyses for Hospital A showed that the AUROC was higher than 0.70 for most subgroups, and net benefit was higher than default strategies at investigated decision thresholds. Patients with ‘ongoing infection at the moment of surgery’ and ‘emergency surgery’ achieved an AUROC of 0.68 (0.65–0.70) and 0.66 (0.61–0.72) respectively and a net benefit not higher than default strategies. The ‘ongoing infection subgroup’ had antibiotics and/or infection treatment before the moment of surgery, but the surgery itself was not to treat an infection. New postoperative infections were labelled in this group if there were at least 24 h between new antibiotics administrations. As both subgroups had a high (40–43%) postoperative infection rate, this could have influenced discriminative performance. Subgroup analyses for Hospitals B and C had a positive net benefit and an AUROC higher than 0.70 for all subgroups. SHAP values show that different features were important for the different hospitals' models, with only ‘High-risk procedure’ being a constant important predictive feature (Fig. 3).

## Discussion

In this multi-centre study, we developed, validated, and updated predictive models within the PERISCOPE AI system to predict postoperative infections within 30- and 7-days post-surgery. Given that observational EHR data may vary by site, it is crucial to investigate local performance in the target population before implementation. To address this domain shift, we used an extensive updating approach with local hospital data and evaluated local models in a temporal setting to ensure stable performance over time. The discriminative performance (mean AUROC) of XGBoost models improved from 0.77–0.86 to 0.81–0.92 after local updating, highlighting the importance of local evaluation and model updates before implementation. Updated models were better calibrated and demonstrated higher clinical utility in terms of net benefit. An extensive subgroup assessment was conducted to identify biases. Variations in incidence rates and performance among different groups underscore the necessity of subgroup assessment before implementing models in a broad patient population.

This is the first study aimed at predicting all types of postoperative infections (including SSIs, pneumoniae, urinary tract infections, and others), using local hospital data for validation that does not require manual data inputs. After external model validation, updating was performed on local historical datasets. Other models predicting postoperative complications e.g., ACS NSQIP and MySurgeryRisk, have limitations as they lack easy integration with the EHR and are not externally validated in other countries and hospital settings.^35^^,^^36^ Our final models outperformed the majority of developed AI models for surgical applications where in 87.5% of the models an AUROC <0.83 was observed in often internal validation settings with small sample sizes.^19^ The need for local updating of AI models has been previously explored.^16^^,^^37^ This so-called ‘myth of generalizability’ advocates for local versions in contrast to the global, off-the-shelf models. This was dramatically demonstrated by the ‘Epic failure’ case of predicting sepsis with a dramatic drop in external model performance.^38^ Our work aligns with recent advancements in the field to allow for local validation and monitoring of AI systems within EHRs and governmental attention to the use of AI in high-risk environments.^39^, ^40^, ^41^, ^42^

This study has some limitations. First, we used a broad definition of infection based on available routinely collected data in the EHR to allow model development and validation without manual labelling of hundreds of thousands of cases. Our definition of postoperative infection partially relies on antibiotics usage, which may need adaptation per hospital setting and site, but allows us to predict the outcome in a broad patient population that does not rely on underreported complications in the EHR.^23^^,^^24^ Second, no thorough fairness evaluation could be performed on ethically sensitive variables such as race and socioeconomic status, due to the unavailability of this information in most European EHRs. Socioeconomic status is known to be a confounder for postoperative infections in Danish arthroplasty patients, indicating the need for predictive models to have accurate performances in these groups.^43^ However, extensive subgroup analysis was performed on other domains and showed stable performance in different types of subgroups. Third, not all clinically relevant variables could be included in model development, including smoking status, due to data unavailability. Using a core set of available features, we were able to create a modelling structure with good predicting capacities, which generalises over different hospital settings and over time. Fourth, while the model demonstrated good overall discrimination through AUROC, the lower AUCPR reflects a limitation in handling imbalanced data, which could affect the precision and recall in real-world clinical settings. The decision curve analysis demonstrated that the model offers clinical utility across a wide range of decision thresholds, confirming its potential applicability in diverse clinical scenarios.

The limited generalizability of models across hospitals may be explained by several factors but are in general attributed to differences in patient demographics and surgery characteristics, and the multivariate distribution between variables captured within EHRs.^44^ This phenomenon of domain shift should be accounted for to achieve local validity and therefore clinical usefulness. We demonstrated that for two academic hospitals with similar incidence rates (Hospital A and Hospital B), discriminative performance significantly improved after model updating with local data. We, therefore, argue that local validation and updating make AI models more suitable to be used in clinical practice. The difference in incidence of postoperative infection rates in Hospital C may be explained by the difference in the type and complexity of procedures performed. However, a lower incidence rate does not necessarily imply that infections have any less clinical impact, as they can still result in severe complications and costly interventions. Furthermore, it may be argued that local model versions result in overfitting, but due to our temporal validation strategies, it was seen that performance was stable over time. Even though optimal model performance should be strived for, updating models requires the availability of a large number of patient records, labour intensive data collection, cleaning and preprocessing, and, once implemented, monitoring per site. Future efforts will be continued to create global models to reduce maintenance and increase robustness, but this requires rigorous data standardisation and data sharing.^45^ Furthermore, dynamic prediction models that handle time-series features postoperatively could be explored in the future to provide updated predictions based on new incoming data. In contrast, we opted for aggregated time-series data in this study, as the current model provides one-time predictions immediately after surgery, and data availability and frequency were inconsistent across the population.

By providing locally valid predictive models as part of the CE-certified PERISCOPE AI system for postoperative infections, we aim to support the early identification of one of the most impactful and costly complications after surgery. Our framework, encompassing model updating, temporal validation, and subgroup analysis across three critical domains—discriminative performance, calibration properties, and clinical utility—can be adapted for various applications to ensure valid prediction models in high-stakes healthcare decision-making contexts. As the actions following the use of the prediction may differ per surgical speciality, e.g., performing additional monitoring or prescribing antibiotics, the relevant decision thresholds to assess clinical usefulness in terms of net benefit will be different per speciality or even per patient.^32^ This means the number of false positives one is willing to accept to find one true positive may vary. We showed that over a wide range of clinically relevant decision thresholds, net benefit was higher for updated models and higher than ‘treat all’ and ‘treat none’ strategies. Furthermore, usability research will be further performed to assess how decision-making is influenced in clinical practice. The PERISCOPE AI system is implemented in local EHRs and therefore does not require manual inputting of data by healthcare professionals. Thorough monitoring of model performance and data drift, i.e., changes in observational data over time, will be performed to ensure quality and safety in daily clinical practice, to adhere to post-market surveillance requirements, and to perform post-market clinical follow-up. Our efforts align with governmental and commercial interest in local validation and monitoring of AI models.^46^^,^^47^ The next steps are to perform clinical trials to investigate PERISCOPE's impact on patient outcomes, decision-making, and costs. PERISCOPE is developed as a ‘Software as a Medical Device’ and is being CE-certified under the Medical Device Regulation (MDR) and efforts to certify under the Food and Drug Administration (FDA) are being initiated.

### Conclusion

In conclusion, we developed and validated PERISCOPE's AI models to accurately predict postoperative infections within 7- and 30-days post-surgery using pre-operative and intra-operative patient data. We demonstrated the importance of local model updating to enhance performance and clinical utility due to differences in patients, procedures, and EHR data. PERISCOPE shows robust performance in predicting postoperative infections, with the potential for safe implementation in varying clinical settings. By conducting this study, we provide a framework for local validation to account for domain shift before implementing AI systems, including updating and performance evaluation in different subgroups.

## Contributors

SLvdM, MSA, BFG, HvG, AMvB, DM, RGHHN, and MGJdB conceptualised and designed the study. HvG, MSA, and DM provided the data. SLvdM, LJS, and MSA conducted data cleaning and analysis. SLvdM drafted the manuscript. All authors reviewed, revised, and edited the manuscript. The PERISCOPE group contributed to the data acquisition, project management, data analyses, and clinical interpretation of the results. All authors reviewed and approved the final manuscript.

## Data sharing statement

Individual participant data, including data dictionaries, will not be available for this study. The analytic code used for creating tables and figures is accessible at gitlab.com/sirivandermeijden/periscope-validation. SLvdM, LJS, and MSA have accessed and verified all data used in the study.

## Declaration of interests

BFG is currently CEO and major shareholder of Healthplus.ai B.V. and subsidiaries. BFG has also received consulting fees from Philips NV and Edwards Lifesciences LLC. SLvdM and LJS are employees at Healthplus.ai B.V. SLvdM and LJS own stock options in Healthplus.ai B.V. HvG is an advisor for Healthplus.ai. MGJdB is the current president of the Dutch foundation for Antimicrobial Policies (Dutch acronym SWAB) and honorary secretary of ESCMID's Study Group on Antimicrobial Policies (ESGAP).

## Appendix ASupplementary data

Supplementary Materials

PERISCOPE Study Group
