# Supplementary material for: Development and validation of artificial intelligence models for early detection of postoperative infections (PERISCOPE): a multicentre study using electronic health record data
Source: Lancet Reg Health Eur. 2024 Dec 5;49:101163. doi: 10.1016/j.lanepe.2024.101163 (PMC11667051; doi:10.1016/j.lanepe.2024.101163)
Supplement: Supplementary Materials [file mmc1.docx]

**Towards Early Detection of Postoperative Infections: Multicentre Development, Validation and Updating of AI Models (PERISCOPE)**

***Supplementary materials***

**Table of Contents**

[1. Procedures inclusion and exclusion 2](#_Toc170305970)

[2. Data description, sample size calculations, pre-processing, and modeling 3](#_Toc170305971)

[2.1. Sample size calculations 3](#_Toc170305972)

[Development](#_Toc170305973) dataset

[Validation](#_Toc170305974) dataset

[2.2. Variables and feature description 4](#_Toc170305975)

[2.3. Outcome definition and labeling strategy 6](#_Toc170305976)

[2.4. Imputation strategies 8](#_Toc170305977)

[2.5. Hyperparameter tuning 9](#_Toc170305978)

[4. PERISCOPE dashboard 9](#_Toc170305979)

[5. Discriminatory performance metrics per hospital 10](#_Toc170305980)

[6. Subgroup analyses per hospital 11](#_Toc170305981)

[References 18](#_Toc170305982)

# Procedures inclusion and exclusion


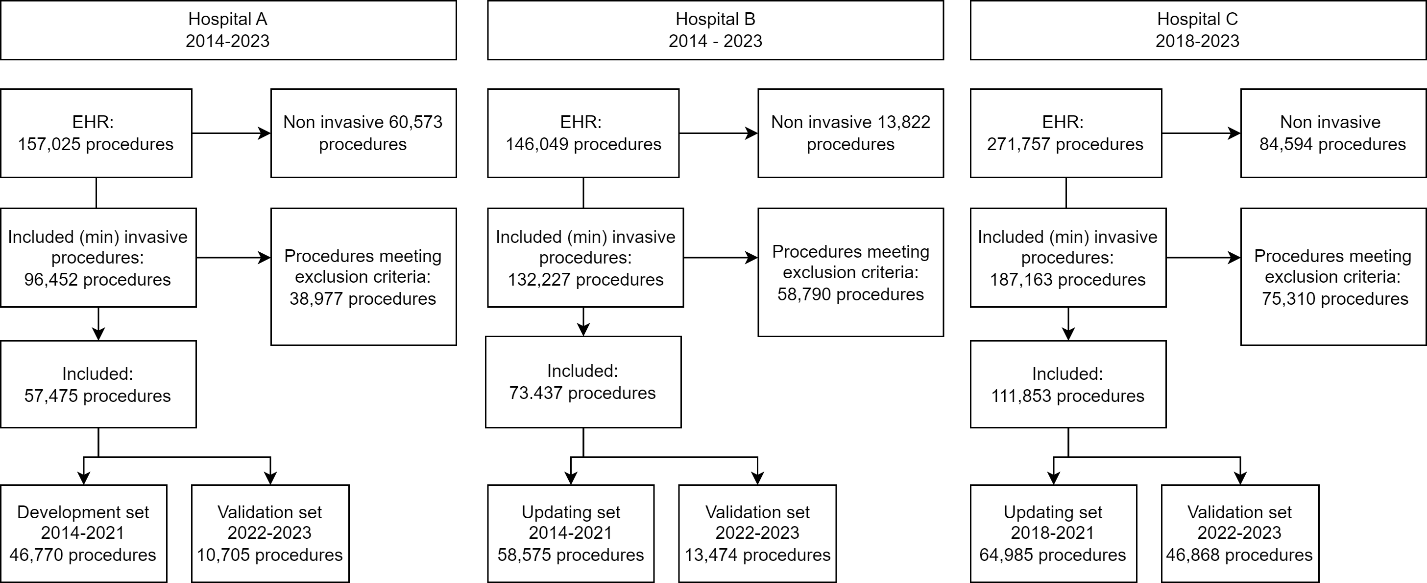


Figure A1: Inclusion and exclusion of procedures per hospital

**Table A1: Visual representation of data per year per hospital datasets. Light grey = data used for development (using 10-fold cross validation) in Hospital A and updating (using 10-fold cross validation) in Hospitals B and C. Dark grey = temporal validation of (updated) models.**

|  | 2014 | 2015 | 2016 | 2017 | 2018 | 2019 | 2020 | 2021 | 2022 | 2023 |
| --- | --- | --- | --- | --- | --- | --- | --- | --- | --- | --- |
| Hospital A |  |  |  |  |  |  |  |  |  |  |
| Hospital B |  |  |  |  |  |  |  |  |  |  |
| Hospital C | N/A | N/A | N/A | N/A |  |  |  |  |  |  |

**Table A2: 30-day mean area under the receiver operating characteristic curve (AUROC) with 95% confidence intervals (CI) per hospital validation dataset (years 2022-2023). These results were generated by alternating the model development site between hospital A, B and C to evaluate whether choosing the development site influences the need for model updating. Grey = local model performance.**

|  | **Hospital A validation dataset AUROC (95% CI)**  (n = 10,705, 14% infection rate) | **Hospital B validation dataset**  **AUROC (95% CI)** (n = 14,180, 14% infection rate) | **Hospital C validation dataset**  **AUROC (95% CI)**  (n = 50,230, 4% infection rate) |
| --- | --- | --- | --- |
| **Model developed on hospital A** | **0.82 (0.81 - 0.83)** | 0.77 (0.76 - 0.79) | 0.85 (0.84 - 0.86) |
| **Model developed on hospital B** | 0.76 (0.74 - 0.77) | **0.82 (0.81 - 0.83)** | 0.70 (0.69 - 0.72) |
| **Model developed on hospital C** | 0.73 (0.72 - 0.75) | 0.72 (0.70 - 0.73) | **0.91 (0.90 - 0.91)** |

# Data description, sample size calculations, pre-processing, and modeling

## 2.1. Sample size calculations

### Development dataset

The generally accepted rule for classical statistical regression analysis is that the training dataset on which the model is developed should have at least 10 events per feature in the dataset to prevent overfitting.^1,2^ In Machine Learning modelling studies, there is no equivalent accepted rule for the minimum sample size, and often no (or post hoc) sample size analysis is done.^3,4^ The rule of thumb of 10 events per variable would require a minimum of 300-1000 events in the training dataset when training a model on 30-100 features. With an infection rate of 10-17% (own data), this would require a minimum of 1,765-10,000 procedures in each development or updating dataset. With the number of surgical patients in each hospital exceeding 10,000 a year (without the exclusion of patients according to section 4.3.), these minimum sample sizes will be achieved for each hospital dataset as we have access to 5+ years of data per hospital.

### Validation dataset

A minimal sample size for reliable assessment of statistical performance should allow at least 100 events for precise estimates of discriminative performance measures and 200 events for estimation of calibration properties.^5-7^ Based on Monte Carlo simulations and sample size calculations, these event rates would allow to detect substantial differences in model performance with 80% power given a 5% fixed type I error.^8^

With an expected outcome rate of 10-17%, the sample size of each assessed dataset, including subgroup analyses, should be at least 589-1,000 procedures to have a precise estimate of discriminative performance and 1,178-2,000 patients to assess calibration properties per subpopulation.

## 2.2. Variables and feature description

**Table A3: Feature descriptions**

|  | **Feature name** | **Description** |
| --- | --- | --- |
| *Vital signs before surgery* (24h before surgery, e.g., mean, std) | |  |
|  | Temperature | Aggregated over time |
|  | Heartrate | Aggregated over time |
|  | Body Mass Index (BMI) | Aggregated over time |
|  | Respiratory rate | Aggregated over time |
|  | Blood pressure | Aggregated over time |
| *Vital signs during surgery* | |  |
|  | FiO2 | Aggregated over time |
|  | Heartrate | Aggregated over time |
|  | Respiratory rate | Aggregated over time |
|  | Blood pressure | Aggregated over time |
|  | SpO2 | Aggregated over time |
| *Lab values before surgery* (24h before surgery, e.g., mean, std) | |  |
|  | ALAT | Aggregated over time |
|  | CRP | Aggregated over time |
|  | Haemoglobin | Aggregated over time |
|  | Leukocytes | Aggregated over time |
| *Procedure* | |  |
|  | specialty_name | Name of the surgical specialty |
|  | procedure_risk¹ | The riskiness of the procedures, based on development or updating data |
|  | body_location² | Body area operated on |
|  | priority | Surgeries priority |
| *Medical history* | |  |
|  | same_day_procs_count | Amount of surgeries undertaken on the day of the current surgery |
|  | past_procedures_count | Amount of surgeries in the six months before surgery |
|  | history_of_uterine_surgery | Uterine surgery present in medical history |
|  | question_asa_score | Indication of patient's overall health |
|  | condition_diabetes | Diabetes diagnose present |
|  | condition_hypertension | Hypertension diagnose present |
|  | condition_infection | Infection diagnosed in the past 6 months |
|  | existing_infection | Infection diagnose active during surgery |
| *Patient* | |  |
|  | sex_male | Patients physician reported sex is male |
|  | age | Patients age |
|  | question_postop_days | Number of days the patient is estimated to be in the hospital for recovery. |
|  | question_preop_days | Number of days the patient has been admitted to the hospital before the surgery. |
| *Medication³* | |  |
|  | medication_immunosuppressant | Immunosuppressant medication administered in the past 6 months |
|  | medication_oral_antidiabetic | Oral antidiabetic medication administered in the past 6 months |
|  | medication_non_oral_antidiab | Non oral antidiabetic medication administered in the past 6 months |
|  | medication_diabetes | Diabetic medication administered in the past 6 months |
|  | medication_diuretics | Diuretics administered in the past 6 months |
|  | medication_beta_blockers | Beta blockers administered in the past 6 months |
|  | medication_ca_chnnl_blockers | Calcium channel blockers administered in the past 6 months |
|  | medication_raas_inhibitors | Renin–angiotensin system medication administered in the past 6 months |
|  | medication_antiarrhythmics | Antiarrhythmic medication administered in the past 6 months |
|  | medication_antithrombotics | Antithrombotic medication administered in the past 6 months |
|  | medication_antineoplastics | Antineoplastic medication administered in the past 6 months |
|  | medication_prednison | Prednisone administered in the past 6 months |
|  | medication_antibiotics | Antibiotic medication administered in the 24 hours before surgery |

1. The feature 'Procedure Risk' was developed to quantify the likelihood of a specific procedure resulting in an infection, using the 30-day postoperative infection as the outcome parameter, similar to a regularized target encoding approach.^15^ The procedure risk feature is calculated based on the procedures in the development or updating dataset. For every procedure that occurs more than 10 times the average infection rate is calculated. Procedures that occurred fewer than 10 times (less than 10% of all procedures) were assigned a 'medium risk' score. This categorization was done to avoid overfitting and potential bias that could arise from using unstable estimates of infection risk from very low-frequency procedures. The top tertile procedures are labeled as ‘high risk’, the bottom tertile as ‘low risk’ and all other procedures - including the procedures that occur less than 10 times - are labeled as ‘medium risk’.

Sensitivity analyses where the threshold of low occurrence was alternated [5, 10, 15, 20] and the default category to which low occurring procedures were assigned to was alternated [low risk, medium risk, high risk] did not lead to significant performance differences. See Tables A4 and A5.

1. Body part is found based on the occurrence of known body part names in the procedure name. Detailed names are grouped into the given categories.
2. Medication is labeled based on the ATC coding system.

**Table A4:** Sensitivity analysis for the ‘Procedure risk’ feature. The threshold value below which procedures were assigned to the default category (Medium risk) based on the occurrence in the development (Hospital A) or updating dataset (Hospital B and C) was varied. Area under the receiver operating characteristic curve (AUROC) was assessed on each hospital’s validation dataset.

| **Threshold value** | **Hospital A AUROC^1^** | **Hospital B AUROC^2^** | **Hospital C AUROC^3^** | **Mean AUROC** |
| --- | --- | --- | --- | --- |
| 5 | 0.816 | 0.819 | 0.908 | 0.864 |
| **10** | 0.816 | 0.819 | 0.908 | 0.864 |
| 15 | 0.815 | 0.817 | 0.909 | 0.863 |
| 20 | 0.818 | 0.816 | 0.910 | 0.863 |

^1^Overall AUROC on hospital A’s validation dataset with 95% confidence intervals was 0.82 (0.81 - 0.83)

^2^Overall AUROC on hospital B’s validation dataset with 95% confidence intervals was 0.82 (0.81 - 0.83)

^3^Overall AUROC on hospital C’s validation dataset with 95% confidence intervals was 0.91 (0.90 - 0.91)

**Table A5:** Sensitivity analysis for the ‘Procedure risk’ feature. The default category (low risk, medium risk or high risk) to which procedures are assigned when they occur less then the threshold value. Area under the receiver operating characteristic curve (AUROC) was assessed on each hospital’s validation dataset.

| **Default value** | **Hospital A AUROC^1^** | **Hospital B AUROC^2^** | **Hospital C AUROC^3^** | **Mean AUROC** |
| --- | --- | --- | --- | --- |
| Low risk | 0.822 | 0.817 | 0.907 | 0.862 |
| **Medium risk** | 0.816 | 0.819 | 0.908 | 0.864 |
| High | 0.813 | 0.818 | 0.900 | 0.859 |

^1^Overall AUROC on hospital A’s validation dataset with 95% confidence intervals was 0.82 (0.81 - 0.83)

^2^Overall AUROC on hospital B’s validation dataset with 95% confidence intervals was 0.82 (0.81 - 0.83)

^3^Overall AUROC on hospital C’s validation dataset with 95% confidence intervals was 0.91 (0.90 - 0.91)

## 2.3. Outcome definition and labeling strategy

Results from literature reviews show that labelling of patients with (postoperative) infections is often done using manual chart review. Manual chart review for identifying postoperative infections in the context of building prediction models is both labor-intensive and prone to errors. The process involves human reviewers (often infection prevention specialists) going through patient electronic health records, extracting relevant data, and interpreting the information to determine whether the patient had an infection.^9^ Furthermore, the accuracy and reliability of manual chart review depends on the expertise and diligence of the reviewer, and it is susceptible to inter-rater variability and errors due to misinterpretation, oversight, or fatigue.^10^ As a result, relying on manual chart review for building prediction models may not only be inefficient but also compromise the quality and generalizability of the models, ultimately affecting their clinical utility and adoption.^11^

To be able to label patients without manual chart review, we worked on establishing a definition that met the following criteria:

1. The criteria should be available in tabular form, i.e., structured, in the EHR databases of at least two hospitals. This means that little pre-processing and no natural language processing on free text and/or image processing is performed.
2. The criteria are focused on detecting patients with all types of bacterial, postoperative infections.
3. The criteria should not only rely on registered complications, as there is a known gap between registration and actual complication rates.

These criteria were agreed upon together with the involved clinicians to allow a scalable (relying on structured EHR data) and broad definition (all types of bacterial infections) to capture as many infections as possible.

The infection definition is made **hospital-specific** for the three criteria (Table A6):

1. **Infection registered:** Based on the diagnosis and complication registration that is used in the hospital, a local list of conditions is determined that they are bacterial infections.
2. **Infection treated with antibiotics:** Based on the medication registration system, all systemic antibiotics relevant for the treatment of postoperative infections are determined (Anatomical Therapeutic Chemical (ATC) codes starting with J01, other coding systems that are converted to ATC codes, or systemic antibiotics determined from a local system converted to ATC codes). Together with the hospital’s infectious disease specialist and/or microbiologist, the prophylactic regimes that should be excluded from the label are determined.

   These are for example:
   1. Gastro-intestinal surgery antibiotic prophylaxis; continued on Cefuroxim **AND** Metronidazol started within 24 hours after surgery up to 5 days.
   2. Asplenic patient prophylaxis;
      1. 1 dd 480 mg Cotrimoxazole (PCP prophylaxis),
      2. **OR** 1 dd 250 mg azithromycin,
      3. **OR** 1 dd 500 mg clarithromycin,
      4. **OR** (1 dd 500mg **or** 2 dd 250mg) amoxicillin (500mg per day in total),
      5. **OR** (1dd 500mg **or** 2dd 250mg) pheneticillin) (500mg per day in total)
   3. Gastroparesis treatment: Low-dose (100 mg) erythromycin
3. **Infection treated with surgical intervention:** Based on the registration names of surgical procedures, it is determined per hospital which are for the treatment of postoperative infections. For example, wound debridement surgery, or drainage of an abscess.

**Table A6. Criteria used to identify patients that had a clinically relevant bacterial, postoperative infection that required 1) registration or 2) treatment based on retrospective electronic health record data. For the prediction within 7 days of surgery, the same definition is used but with a time cut-off of 7 days instead of 30.**

| **#** | **Criterion** | **Condition** | **Explanation** |
| --- | --- | --- | --- |
| 1 | Infection registered | Infection is registered by a surgeon by means of a specific condition (ICD code or hospital-specific code) AND the condition onset date is in between the next calendar day after surgery and before 30 days after surgery. | Infections are registered in the Electronic Health Record by the surgeon or resident using either ICD-9/10/11 codes^12^ or a hospital/department-specific complication registration system. Registration is often performed at the moment of discharge or during department-wide meetings to go over all complications. Note that the registration is most often not performed at the moment the infection started and that there is large under-registration of complications in general^13,14^. |
| **AND/OR** | | | |
| 2 | Infection treated with antibiotics | Patient received antibiotic treatment between >= 24 hours and <= 30 days after surgery, with a duration longer than 3 days. BUT NOT extended (beyond 24 hours after surgery) prophylaxis and non-infection related regimens* | Infections are treated with antibiotics. Some patients (depending on surgery type and patient history) will receive prophylactic antibiotics in the period before, or directly after surgery. To exclude prophylactic antibiotics, treatment has to start after >= 24 h after surgery. Other specific (gastro-intestinal and asplenic) prophylactic regimes are excluded as well. Some patients will receive gastroparesis treatment in the form of erythromycin, which is not to treat a bacterial infection.  Sometimes, an infection is suspected for which antibiotics is started, but cultures may come back negative. To exclude these cases, the minimum duration is set at 3 days. |
| **AND/OR** | | | |
| 3 | Infection treated with surgical intervention | Patient received a surgical intervention related to treatment of infection within 0 days < surgery < 30 days BUT NOT during initial surgery itself. | (Deep) surgical site infections sometimes require repeated surgery to drain the infection and/or clean the wound. These treatments are done at the operating room and are therefore registered as surgical procedures. The different types of surgical procedures performed at each hospital are filtered on treatments related to postoperative infections. |

*Prophylactic regimes that are excluded are hospital specific.

## 2.4. Imputation strategies

For missing values, an analysis was performed per hospital’s development or updating dataset to check availability and correlation with the outcome. XGBoost can handle missing data but specific imputation strategies were applied in some cases. Features that were more than 80% missing were not included (except for laboratory values due to missingness not at random). Features without a correlation to the predicted outcome (difference in average infection with or without value less than 15%) were imputed with the median. For features with a significant correlation to the predicted outcome, a strategy was chosen according to these rules:

If the infection rate with missing values was lower than the infection rate without missing values, it is assumed that the value will be missing for patients in a better condition (eg: lab values will be ordered less often for healthier patients). In this case, either the signal (values are missing) is conserved by leaving the value missing (this option has been opted for lab values) or the missing value has been imputed with a normal (healthy) value (e.g., 12 /min for respiratory rate).

If the infection rate was increased for missing values, this signal was conserved and the values were left missing.

## 2.5. Hyperparameter tuning

For each hospital the hyperparameters for XGBoost were optimized with a 200 iteration random grid search. See Table A3 for the explored hyperparameter space.

**Table A7: Hyperparameter space for XGBoost**

| Parameter | Range | Distribution |
| --- | --- | --- |
| Max_depth | Integer: [2,12] | Uniform |
| N_estimators | Integer: [5,1000] | Uniform |
| Gamma | Float: (0,1] | Uniform |
| Colsample_bytree | Float: [0.3,1] | Uniform |
| Subsample | Float: [0.3,1] | Uniform |
| reg_alpha | Float: [0.01,100] | Logarithmic |
| reg_lambda | Float: [0.01,100] | Logarithmic |
| min_child_weight | Float: [0,1000] | Uniform |

# PERISCOPE dashboard


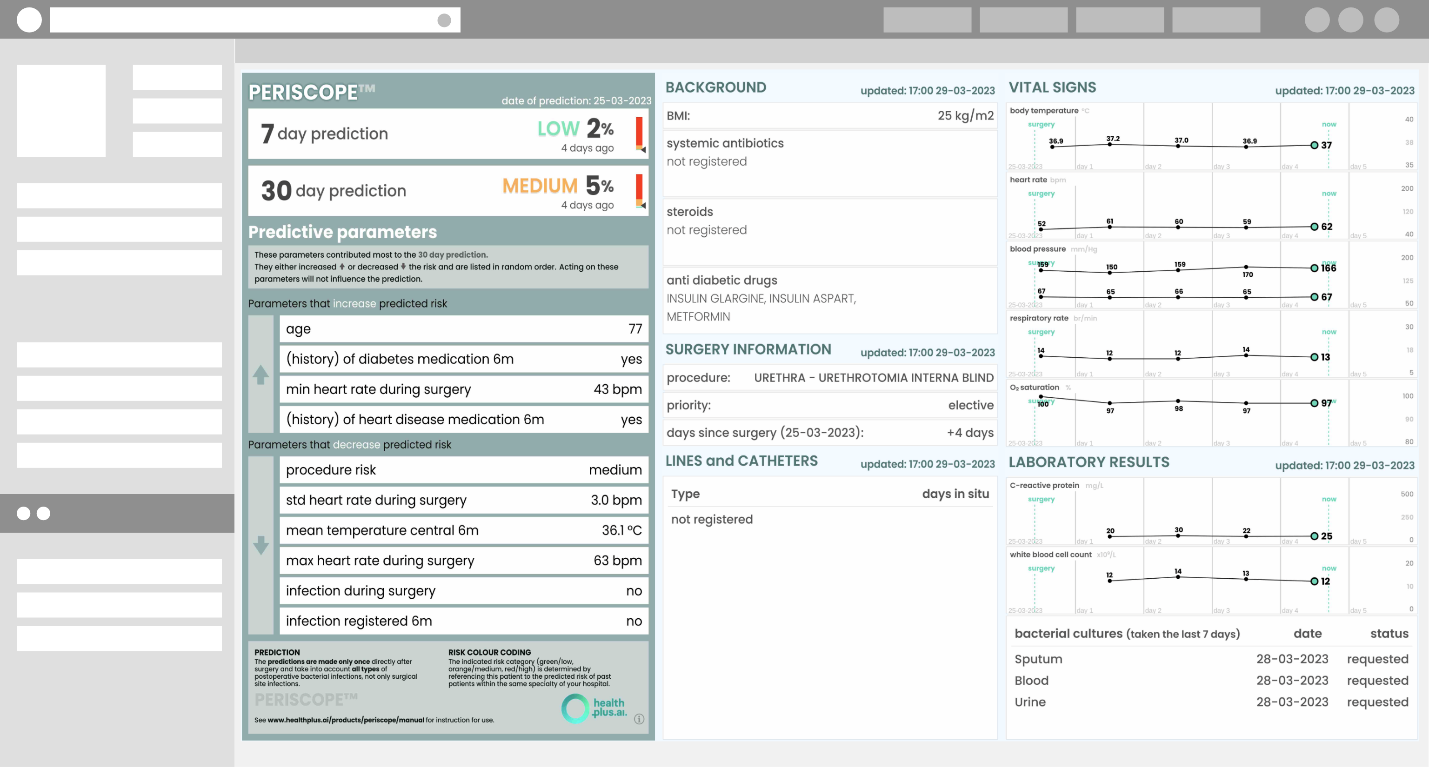


**Figure A2: PERISCOPE dashboard of a fictive patient. Risk categories low, medium, or high are determined per surgical specialty based on the postoperative infection incidence rate.**

# Discriminatory performance metrics per hospital

**Table A8:** All discriminative performance and calibration metrics for Leiden University Medical Center (LUMC). Cut-off sensitive metrics (accuracy, precision, NPV, recall, F1 score (harmonic mean of PPV and sensitivity)) are calculated for the cut-off equal to the infection rate in the development datasets. CI = confidence interval, AUROC = area under the receiver operating characteristic curve, AUCPR = area under the precision recall curve, CV = cross-validation, NPV = negative predictive value, PPV = positive predictive value, SD = standard deviation

| Metric | Internal CV on development dataset (mean +- SD) | Temporal validation dataset (95% CI) | Internal CV on development dataset (mean +- SD) | Temporal validation dataset (95% CI) |
| --- | --- | --- | --- | --- |
| Prediction timeframe | < 30 days | | < 7 days | |
| **Infection rate (%)** | 15% | 14% |  | 8% |
| **Accuracy** | 0.77 (0.01) | 0.74 (0.73-0.75) | 0.76 (0.01) | 0.75 (0.74-0.75) |
| **Precision (PPV)** | 0.36 (0.01) | 0.32 (0.30-0.33) | 0.23 (0.01) | 0.19 (0.18-0.21) |
| **Specificity** | 0.77 (0.01) | 0.74 (0.73-0.75) | 0.76 (0.01) | 0.75 (0.74-0.75) |
| **NPV** | 0.95 (0.01) | 0.95 (0.94-0.95) | 0.97 (0.00) | 0.97 (0.97-0.97) |
| **Recall (sensitivity)** | 0.76 (0.03) | 0.75 (0.73-0.77) | 0.77 (0.02) | 0.72 (0.70-0.75) |
| **F1-score** | 0.49 (0.01) | 0.45 (0.43-0.46) | 0.36 (0.01) | 0.31 (0.29-0.32) |
| **AUROC** | 0.83 (0.01) | 0.82 (0.81-0.83) | 0.83 (0.01) | 0.81 (0.80-0.82) |
| **AUCPR** | 0.49 (0.03) | 0.43 (0.41-0.46) | 0.33 (0.02) | 0.26 (0.23-0.28) |
| **Brier score** | 0.10 (0.00) | 0.10 (0.09-0.10) | 0.07 (0.00) | 0.06 (0.06-0.07) |

**Table A9:** All discriminative performance and calibration metrics for Radboud University Medical Center (Radboud UMC). Cut-off sensitive metrics (accuracy, precision, NPV, recall, F1 score (harmonic mean of PPV and sensitivity)) are calculated for the cut-off equal to the infection rate in the updating datasets. CI = confidence interval, AUROC = area under the receiver operating characteristic curve, AUCPR = area under the precision recall curve, CV = cross-validation, NPV = negative predictive value, PPV = positive predictive value, SD = standard deviation

| Metric | Internal CV on updating dataset (mean +- SD) | Temporal validation dataset (95% CI) | Internal CV on updating dataset (mean +- SD) | Temporal validation dataset (95% CI) |
| --- | --- | --- | --- | --- |
| Prediction timeframe | < 30 days | | < 7 days | |
| **Infection rate (%)** | 15% | 14% | 7% | 7% |
| **Accuracy** | 0.79 (0.00) | 0.78 (0.78-0.79) | 0.78 (0.00) | 0.77 (0.77-0.78) |
| **Precision (PPV)** | 0.36 (0.01) | 0.36 (0.35-0.37) | 0.20 (0.01) | 0.20 (0.19-0.21) |
| **Specificity** | 0.80 (0.00) | 0.80 (0.79-0.81) | 0.79 (0.01) | 0.78 (0.77-0.78) |
| **NPV** | 0.94 (0.00) | 0.94 (0.94-0.94) | 0.97 (0.00) | 0.97 (0.97-0.98) |
| **Recall (sensitivity)** | 0.69 (0.02) | 0.68 (0.67-0.70) | 0.72 (0.03) | 0.71 (0.69-0.74) |
| **F1-score** | 0.47 (0.01) | 0.47 (0.46-0.49) | 0.32 (0.01) | 0.31 (0.29-0.32) |
| **AUROC** | 0.83 (0.01) | 0.82 (0.81-0.83) | 0.83 (0.01) | 0.81 (0.80-0.83) |
| **AUCPR** | 0.54 (0.02) | 0.51 (0.49-0.53) | 0.33 (0.03) | 0.32 (0.29-0.34) |
| **Brier score** | 0.09 (0.00) | 0.09 (0.09-0.09) | 0.05 (0.00) | 0.05 (0.05-0.06) |

**Table A10: All discriminative performance and calibration metrics for ZOL Genk. Cut-off sensitive metrics (accuracy, precision, NPV, recall, F1 score (harmonic mean of PPV and sensitivity)) are calculated for the cut-off equal to the infection rate in the updating datasets. CI = confidence interval, AUROC = area under the receiver operating characteristic curve, AUCPR = area under the precision recall curve, CV = cross-validation, NPV = negative predictive value, PPV = positive predictive value, SD = standard deviation**

| Metric | Internal CV on updating dataset (mean +- SD) | Temporal validation dataset (95% CI) | Internal CV on updating dataset (mean +- SD) | Temporal validation dataset (95% CI) |
| --- | --- | --- | --- | --- |
| Prediction timeframe | < 30 days | | < 7 days | |
| **Infection rate (%)** | 4% | 4% | 3% | 3% |
| **Accuracy** | 0.84 (0.01) | 0.86 (0.86-0.86) | 0.85 (0.01) | 0.87 (0.87-0.87) |
| **Precision (PPV)** | 0.19 (0.01) | 0.19 (0.18-0.19) | 0.15 (0.01) | 0.14 (0.14-0.15) |
| **Specificity** | 0.84 (0.01) | 0.86 (0.86-0.86) | 0.85 (0.01) | 0.87 (0.87-0.87) |
| **NPV** | 0.99 (0.00) | 0.99 (0.99-0.99) | 0.99 (0.00) | 0.99 (0.99-1.00) |
| **Recall (sensitivity)** | 0.85 (0.02) | 0.82 (0.80-0.84) | 0.84 (0.03) | 0.82 (0.80-0.84) |
| **F1-score** | 0.32 (0.01) | 0.30 (0.29-0.32) | 0.25 (0.01) | 0.24 (0.23-0.25) |
| **AUROC** | 0.91 (0.01) | 0.91 (0.90-0.91) | 0.91 (0.01) | 0.92 (0.91-0.92) |
| **AUCPR** | 0.39 (0.02) | 0.37 (0.35-0.40) | 0.29 (0.03) | 0.29 (0.27-0.31) |
| **Brier score** | 0.03 (0.00) | 0.03 (0.03-0.03) | 0.02 (0.00) | 0.02 (0.02-0.02) |

# Subgroup analyses per hospital

**Table A11: Subgroup analysis evaluation of the final model on the** validation **dataset of hospital A. ASAP = as soon as possible, AUROC = area under the receiver operating characteristic curve, CI = confidence interval.**

| **Group** | **Subgroup** | **Number of patient (% of total validation set)** | **Outcome, n (%)** | **AUROC (95% CI)** | **Calibration slope (95% CI)** | **Calibration intercept (95% CI)** | **Delta net benefit > 0** |
| --- | --- | --- | --- | --- | --- | --- | --- |
| **Sex** | Female | 5708 (53.3) | 631 (11.1) | 0.82 (0.81-0.84) | 0.93 (0.86-0.99) | -0.20 (-0.26--0.11) | TRUE |
|  | Male | 4997 (46.7) | 858 (17.2) | 0.80 (0.79-0.82) | 0.85 (0.79-0.92) | -0.01 (-0.10-0.07) | TRUE |
| **Age** | 18-40 | 2217 (20.7) | 229 (10.3) | 0.86 (0.84-0.88) | 1.02 (0.92-1.13) | -0.05 (-0.18-0.08) | TRUE |
|  | 40-60 | 3520 (32.9) | 429 (12.2) | 0.81 (0.79-0.84) | 0.86 (0.80-0.96) | -0.12 (-0.24-0.00) | TRUE |
|  | >60 | 4968 (46.4) | 831 (16.7) | 0.79 (0.78-0.81) | 0.87 (0.80-0.93) | -0.09 (-0.17--0.01) | TRUE |
| **Emergency** | ASAP | 1848 (17.3) | 511 (27.7) | 0.76 (0.74-0.78) | 0.78 (0.70-0.87) | -0.08 (-0.16-0.00) | TRUE |
|  | Elective | 8547 (79.8) | 854 (10.0) | 0.80 (0.79-0.81) | 0.91 (0.86-0.96) | -0.13 (-0.19--0.05) | TRUE |
|  | Emergency | 310 (2.9) | 124 (40.0) | 0.66 (0.61-0.72) | 0.58 (0.37-0.82) | 0.18 (-0.06-0.42) | FALSE |
| **Specialty** | Otorhinolaryngology | 1578 (14.7) | 129 (8.2) | 0.83 (0.80-0.87) | 0.92 (0.80-1.03) | -0.08 (-0.23-0.11) | TRUE |
|  | General surgery | 4225 (39.5) | 883 (20.9) | 0.79 (0.78-0.80) | 0.81 (0.76-0.88) | -0.04 (-0.11-0.05) | TRUE |
|  | Gyneacology | 990 (9.2) | 71 (7.2) | 0.75 (0.71-0.79) | 0.80 (0.64-0.99) | -0.24 (-0.44--0.00) | TRUE |
|  | Jaw surgery | 354 (3.3) | 15 (4.2) | 0.82 (0.72-0.91) | 1.07 (0.72-1.46) | -0.19 (-0.70-0.31) | TRUE |
|  | Neurosurgery | 1694 (15.8) | 185 (10.9) | 0.77 (0.74-0.81) | 0.88 (0.77-1.02) | -0.22 (-0.38--0.06) | TRUE |
|  | Orthopaedic surgery | 965 (9.0) | 97 (10.1) | 0.85 (0.81-0.88) | 1.09 (0.93-1.27) | -0.30 (-0.48--0.10) | TRUE |
|  | Plastic surgery | 272 (2.5) | 20 (7.4) | 0.95 (0.92-0.98) | 1.89 (1.40-2.54) | -0.61 (-0.99--0.25) | TRUE |
|  | Urology | 627 (5.9) | 89 (14.2) | 0.75 (0.71-0.79) | 0.97 (0.77-1.16) | 0.15 (-0.09-0.36) | TRUE |
| **Surgical procedure location** | Abdomen | 2752 (25.7) | 687 (25.0) | 0.74 (0.72-0.76) | 0.76 (0.68-0.85) | -0.08 (-0.19-0.01) | TRUE |
|  | Brain | 739 (6.9) | 80 (10.8) | 0.73 (0.69-0.78) | 0.72 (0.60-0.86) | -0.05 (-0.31-0.19) | TRUE |
|  | Central nervous system | 152 (1.4) | 19 (12.5) | 0.79 (0.70-0.88) | 1.05 (0.66-1.55) | -0.16 (-0.60-0.30) | TRUE |
|  | Extremity | 1840 (17.2) | 197 (10.7) | 0.87 (0.84-0.89) | 1.09 (0.98-1.22) | -0.04 (-0.19-0.10) | TRUE |
|  | Genitals | 844 (7.9) | 49 (5.8) | 0.76 (0.72-0.82) | 0.90 (0.72-1.15) | -0.09 (-0.35-0.12) | TRUE |
|  | Head | 2049 (19.1) | 139 (6.8) | 0.79 (0.75-0.83) | 0.86 (0.74-1.00) | -0.06 (-0.25-0.11) | TRUE |
|  | Hip | 304 (2.8) | 57 (18.8) | 0.76 (0.70-0.82) | 0.90 (0.66-1.16) | -0.02 (-0.31-0.33) | TRUE |
|  | Other | 1741 (16.3) | 190 (10.9) | 0.82 (0.78-0.85) | 0.94 (0.79-1.07) | -0.27 (-0.44--0.11) | TRUE |
|  | Thorax | 273 (2.6) | 70 (25.6) | 0.77 (0.72-0.82) | 1.09 (0.86-1.39) | -0.04 (-0.33-0.22) | TRUE |
| **Ongoing infection at the moment of surgery** | No | 9546 (89.2) | 997 (10.4) | 0.79 (0.78-0.80) | 0.89 (0.85-0.94) | -0.07 (-0.13--0.00) | TRUE |
|  | Yes | 1159 (10.8) | 492 (42.5) | 0.68 (0.65-0.70) | 0.80 (0.69-0.95) | -0.18 (-0.30--0.06) | FALSE |

**Table A12: Subgroup analysis of the final model (updated) on the validation dataset of Radboud UMC**

| **Group** | **Subgroup** | **Number of patient (% of total validation set)** | **Outcome, n (%)** | **AUROC (95% CI)** | **Calibration slope (95% CI)** | **Calibration intercept (95% CI)** | **Delta net benefit > 0** |
| --- | --- | --- | --- | --- | --- | --- | --- |
| **Sex** | Female | 6503 (48.3) | 790 (12.1) | 0.81 (0.79-0.83) | 0.96 (0.91-1.02) | -0.09 (-0.15--0.02) | TRUE |
|  | Male | 6971 (51.7) | 1197 (17.2) | 0.81 (0.80-0.83) | 0.93 (0.87-0.98) | -0.05 (-0.12-0.02) | TRUE |
| **Age** | 18-40 | 2722 (20.2) | 340 (12.5) | 0.82 (0.79-0.84) | 0.96 (0.89-1.05) | 0.05 (-0.07-0.17) | TRUE |
|  | 40-60 | 3993 (29.6) | 570 (14.3) | 0.83 (0.81-0.85) | 0.98 (0.90-1.05) | -0.09 (-0.18-0.02) | TRUE |
|  | >60 | 6759 (50.2) | 1077 (15.9) | 0.80 (0.79-0.82) | 0.93 (0.88-0.98) | -0.08 (-0.14--0.01) | TRUE |
| **Emergency** | ASAP | 1549 (11.5) | 480 (31.0) | 0.82 (0.80-0.84) | 0.94 (0.87-1.02) | 0.09 (-0.01-0.21) | TRUE |
|  | Elective | 11571 (85.9) | 1373 (11.9) | 0.78 (0.77-0.80) | 0.89 (0.86-0.94) | -0.11 (-0.17--0.04) | TRUE |
|  | Emergency | 354 (2.6) | 134 (37.9) | 0.83 (0.79-0.87) | 1.12 (0.91-1.35) | 0.06 (-0.14-0.24) | TRUE |
| **Specialty** | Otorhinolaryngology | 1260 (9.4) | 92 (7.3) | 0.76 (0.72-0.80) | 0.89 (0.72-1.06) | -0.17 (-0.39-0.06) | TRUE |
|  | General surgery | 2040 (15.1) | 635 (31.1) | 0.80 (0.78-0.82) | 0.93 (0.85-1.04) | -0.01 (-0.13-0.11) | TRUE |
|  | Gynaecology | 1455 (10.8) | 94 (6.5) | 0.69 (0.65-0.74) | 0.79 (0.62-0.98) | -0.17 (-0.39--0.01) | TRUE |
|  | Jaw surgery | 1034 (7.7) | 125 (12.1) | 0.70 (0.66-0.75) | 0.78 (0.60-0.99) | 0.26 (0.09-0.44) | TRUE |
|  | Neurosurgery | 1643 (12.2) | 197 (12.0) | 0.88 (0.86-0.90) | 1.26 (1.16-1.38) | -0.06 (-0.19-0.11) | TRUE |
|  | Orthopaedic surgery | 1945 (14.4) | 228 (11.7) | 0.82 (0.80-0.85) | 0.85 (0.76-0.95) | -0.28 (-0.40--0.16) | TRUE |
|  | Cardiothoracic surgery | 2015 (15.0) | 288 (14.3) | 0.83 (0.80-0.85) | 1.04 (0.95-1.17) | -0.25 (-0.41--0.12) | TRUE |
|  | Urology | 2082 (15.5) | 328 (15.8) | 0.71 (0.68-0.74) | 0.78 (0.66-0.91) | 0.13 (0.03-0.26) | TRUE |
| **Surgical procedure location** | Abdomen | 2396 (17.8) | 567 (23.7) | 0.80 (0.78-0.82) | 0.89 (0.81-0.96) | -0.05 (-0.17-0.05) | TRUE |
|  | Brain | 1358 (10.1) | 158 (11.6) | 0.88 (0.86-0.91) | 1.24 (1.11-1.41) | -0.08 (-0.23-0.08) | TRUE |
|  | Central nervous system | 115 (0.9) | 11 (9.6) | 0.93 (0.81-1.00) | 2.50 (1.40-3.82) | -0.11 (-0.73-0.36) | TRUE |
|  | Extremity | 1632 (12.1) | 235 (14.4) | 0.82 (0.79-0.84) | 0.84 (0.76-0.93) | -0.04 (-0.19-0.08) | TRUE |
|  | Genitals | 1506 (11.2) | 119 (7.9) | 0.70 (0.66-0.74) | 0.78 (0.64-0.94) | -0.01 (-0.21-0.19) | TRUE |
|  | Head | 2039 (15.1) | 190 (9.3) | 0.75 (0.72-0.78) | 0.90 (0.78-1.06) | 0.12 (-0.02-0.29) | TRUE |
|  | Hip | 761 (5.6) | 114 (15.0) | 0.85 (0.81-0.88) | 0.96 (0.81-1.14) | -0.32 (-0.54--0.10) | TRUE |
|  | Other | 1517 (11.3) | 271 (17.9) | 0.75 (0.73-0.79) | 0.89 (0.79-1.03) | -0.01 (-0.15-0.14) | TRUE |
|  | Thorax | 2150 (16.0) | 322 (15.0) | 0.82 (0.80-0.85) | 1.05 (0.94-1.14) | -0.22 (-0.33--0.07) | TRUE |
| **Ongoing infection at the moment of surgery** | No | 11750 (87.2) | 1180 (10.0) | 0.75 (0.73-0.76) | 0.91 (0.86-0.97) | -0.08 (-0.13--0.03) | TRUE |
|  | Yes | 1724 (12.8) | 807 (46.8) | 0.77 (0.75-0.79) | 0.85 (0.77-0.94) | -0.01 (-0.10-0.10) | FALSE |

**Table A13: Subgroup analysis of the final model (updated) on the validation dataset of ZOL Genk**

| **Group** | **Subgroup** | **Number of patient (% of total validation set)** | **Outcome, n ( %)** | **AUROC (95% CI)** | **Calibration slope (95% CI)** | **Calibration intercept (95% CI)** | **Delta net benefit > 0** |
| --- | --- | --- | --- | --- | --- | --- | --- |
| **Sex** | Female | 24657 (52.6) | 727 (2.9) | 0.90 (0.89-0.91) | 1.01 (0.97-1.05) | 0.10 (0.02-0.17) | TRUE |
|  | Male | 22211 (47.4) | 901 (4.1) | 0.91 (0.90-0.92) | 0.96 (0.93-1.00) | 0.03 (-0.05-0.10) | TRUE |
| **Age** | 18-40 | 11074 (23.6) | 157 (1.4) | 0.89 (0.86-0.92) | 1.10 (1.02-1.20) | 0.22 (0.10-0.34) | TRUE |
|  | 40-60 | 16084 (34.3) | 390 (2.4) | 0.90 (0.88-0.91) | 0.99 (0.93-1.04) | -0.01 (-0.11-0.11) | TRUE |
|  | >60 | 19710 (42.1) | 1081 (5.5) | 0.89 (0.88-0.90) | 0.95 (0.92-0.99) | 0.07 (0.01-0.13) | TRUE |
| **Emergency** | ASAP | 3935 (8.4) | 463 (11.8) | 0.86 (0.84-0.87) | 0.86 (0.81-0.93) | 0.18 (0.08-0.29) | TRUE |
|  | Elective | 42185 (90.0) | 982 (2.3) | 0.89 (0.88-0.90) | 1.00 (0.97-1.04) | 0.01 (-0.05-0.08) | TRUE |
|  | Emergency | 748 (1.6) | 183 (24.5) | 0.83 (0.80-0.86) | 0.83 (0.71-0.97) | 0.16 (-0.06-0.35) | TRUE |
| **Specialty** | Otorhinolaryngology | 2598 (5.5) | 18 (0.7) | 0.90 (0.81-0.96) | 1.18 (0.93-1.55) | -0.51 (-1.00--0.11) | TRUE |
|  | General surgery | 10019 (21.4) | 642 (6.4) | 0.89 (0.88-0.91) | 0.96 (0.91-1.01) | 0.14 (0.04-0.24) | TRUE |
|  | Gynaecology | 3304 (7.0) | 33 (1.0) | 0.89 (0.84-0.95) | 1.29 (1.09-1.52) | 0.09 (-0.36-0.36) | TRUE |
|  | Jaw surgery | 2390 (5.1) | 37 (1.5) | 0.89 (0.82-0.95) | 1.15 (0.96-1.37) | 0.29 (0.04-0.57) | TRUE |
|  | Neurosurgery | 4386 (9.4) | 217 (4.9) | 0.91 (0.90-0.93) | 1.06 (0.98-1.16) | -0.14 (-0.27--0.01) | TRUE |
|  | Orthopaedic surgery | 18409 (39.3) | 386 (2.1) | 0.88 (0.86-0.90) | 0.96 (0.91-1.01) | 0.09 (0.00-0.19) | TRUE |
|  | Plastic surgery | 1783 (3.8) | 22 (1.2) | 0.79 (0.68-0.88) | 0.87 (0.61-1.16) | -0.23 (-0.63-0.10) | TRUE |
|  | Cardiothoracic surgery | 700 (1.5) | 192 (27.4) | 0.67 (0.63-0.72) | 0.90 (0.70-1.14) | -0.04 (-0.20-0.11) | TRUE |
|  | Urology | 3279 (7.0) | 81 (2.5) | 0.85 (0.81-0.89) | 0.97 (0.84-1.12) | 0.13 (-0.15-0.38) | TRUE |
| **Surgical procedure location** | Abdomen | 2224 (4.7) | 74 (3.3) | 0.82 (0.78-0.88) | 1.10 (0.91-1.31) | 0.23 (-0.03-0.47) | TRUE |
|  | Brain | 47 (0.1) | 1 (2.1) | 0.98 (0.95-nan) | 54.05 (-0.00-275.02) | -10.04 (-21.55--0.57) | TRUE |
|  | Extremity | 12811 (27.3) | 243 (1.9) | 0.87 (0.85-0.89) | 1.00 (0.91-1.10) | 0.10 (-0.00-0.24) | TRUE |
|  | Genitals | 918 (2.0) | 10 (1.1) | 0.80 (0.66-0.93) | 1.16 (0.79-1.59) | -0.33 (-1.09-0.19) | TRUE |
|  | Head | 1119 (2.4) | 27 (2.4) | 0.90 (0.83-0.95) | 0.87 (0.70-1.09) | -0.65 (-0.97--0.27) | TRUE |
|  | Hip | 750 (1.6) | 75 (10.0) | 0.72 (0.68-0.77) | 0.50 (0.39-0.64) | 0.00 (-0.27-0.25) | TRUE |
|  | Other | 28505 (60.8) | 1078 (3.8) | 0.92 (0.91-0.93) | 1.01 (0.98-1.04) | 0.09 (0.03-0.15) | TRUE |
|  | Thorax | 494 (1.1) | 120 (24.3) | 0.63 (0.58-0.68) | 0.77 (0.38-1.13) | -0.08 (-0.28-0.11) | TRUE |
| **Ongoing infection at the moment of surgery** | No | 45129 (96.3) | 1137 (2.5) | 0.88 (0.87-0.90) | 0.99 (0.95-1.03) | 0.03 (-0.04-0.10) | TRUE |
|  | Yes | 1739 (3.7) | 491 (28.2) | 0.83 (0.81-0.85) | 0.79 (0.71-0.87) | 0.16 (0.04-0.29) | TRUE |

# References

1. Vittinghoff E, McCulloch CE. Relaxing the rule of ten events per variable in logistic and Cox regression. Am J Epidemiol. 2007;165(6):710-718.
2. Shmueli G. To explain or to predict? Stat Sci. 2010;25(3):289-310.
3. de Hond AAH, Leeuwenberg AM, Hooft L, Kant IMJ, Nijman SWJ, van Os HJA, et al. Guidelines and quality criteria for artificial intelligence-based prediction models in healthcare: a scoping review. NPJ Digit Med. 2022;5(1):1-13.
4. Balki I, Amirabadi A, Levman J, Martel AL, Emersic Z, Meden B, et al. Sample-size determination methodologies for machine learning in medical imaging research: a systematic review. Can Assoc Radiol J. 2019;70(4):344-353.
5. Collins GS, Ogundimu EO, Altman DG. Sample size considerations for the external validation of a multivariable prognostic model: a resampling study. Stat Med. 2016;35(2):214-226.
6. Archer L, Snell KIE, Ensor J, Hudda MT, Collins GS, Riley RD. Minimum sample size for external validation of a clinical prediction model with a continuous outcome. Stat Med. 2021;40:133-146.
7. Riley RD, Debray TPA, Collins GS, Archer L, Ensor J, van Smeden M, Snell KIE. Minimum sample size for external validation of a clinical prediction model with a binary outcome. Stat Med. 2021;40(19):4230-4251.
8. Vergouwe Y, Steyerberg EW, Eijkemans MJ, Habbema JD. Substantial effective sample sizes were required for external validation studies of predictive logistic regression models. J Clin Epidemiol. 2005;58(5):475-483.
9. Kuhn T, Basch P, Barr M, Yackel T. Clinical documentation in the 21st century: executive summary of a policy position paper from the American College of Physicians. Ann Intern Med. 2015;162(4):301-303.
10. Vassar M, Holzmann M. The retrospective chart review: important methodological considerations. J Educ Eval Health Prof. 2013;10:12.
11. Murff HJ, FitzHenry F, Matheny ME, Gentry N, Kotter KL, Crimin K, et al. Automated identification of postoperative complications within an electronic medical record using natural language processing. JAMA. 2011;306(8):848-855.
12. World Health Organization. ICD-10: international statistical classification of diseases and related health problems: tenth revision, 2nd ed. Geneva: World Health Organization; 2004.
13. Ubbink DT, Visser A, Gouma DJ, Goslings JC. Registration of surgical adverse outcomes: a reliability study in a university hospital. BMJ Open. 2012;2(3)
14. Veen EJ, Janssen-Heijnen ML, Bosma E, de Jongh MA, Roukema JA. The accuracy of complications documented in a prospective complication registry. J Surg Res. 2012;173(1):54-59.
15. Pargent F, Pfisterer F, Thomas J, Bischl B. Regularized target encoding outperforms traditional methods in supervised machine learning with high cardinality features. *Computational Statistics* 2022; **37**(5): 2671-92.
