# Supplementary material for: Development and validation of artificial intelligence models for early detection of postoperative infections (PERISCOPE): a multicentre study using electronic health record data
Source: Lancet Reg Health Eur. 2024 Dec 5;49:101163. doi: 10.1016/j.lanepe.2024.101163 (PMC11667051; doi:10.1016/j.lanepe.2024.101163)
Supplement: PERISCOPE Study Group [file mmc2.docx]

**PERISCOPE study group**

| **First Name** | **Surname** | **Site** |
| --- | --- | --- |
| Pieter | de Heer | Rigshospitalet |
| Jaap | Hamming | Leiden University Medical Centre |
| Karin Ellen | Veldkamp | Leiden University Medical Centre |
| Wilco | Peul | Leiden University Medical Centre |
| Rolv-Ole | Lindsetmo | University Hospital of North Norway |
| Maxime | Kummeling | Leiden University Medical Centre |
| Jogchum | Beltman | Leiden University Medical Centre |
| Merlijn | Hutteman | Radboud University Medical Centre |
| Alma | Tostman | Radboud University Medical Centre |
| Wim | Reijnen | Radboud University Medical Centre |
| Bas | Bredie | Radboud University Medical Centre |
| Ilse | Spenkelink | Radboud University Medical Centre |
| Ben | Goethuys | Hospital Oost Limburg |
| Noëlla | Pierlet | Hospital Oost Limburg |
| Joost | Huiskens | Microsoft |
